# A FAIR guide for data providers to maximise sharing of human genomic data

**DOI:** 10.1371/journal.pcbi.1005873

**Published:** 2018-03-15

**Authors:** Manuel Corpas, Nadezda V. Kovalevskaya, Amanda McMurray, Fiona G. G. Nielsen

**Affiliations:** Repositive Ltd, Betjeman House, Cambridge, United Kingdom; Genome Quebec, CANADA

## Abstract

It is generally acknowledged that, for reproducibility and progress of human genomic research, data sharing is critical. For every sharing transaction, a successful data exchange is produced between a data consumer and a data provider. Providers of human genomic data (e.g., publicly or privately funded repositories and data archives) fulfil their social contract with data donors when their shareable data conforms to FAIR (findable, accessible, interoperable, reusable) principles. Based on our experiences via Repositive (https://repositive.io), a leading discovery platform cataloguing all shared human genomic datasets, we propose guidelines for data providers wishing to maximise their shared data’s FAIRness.

## Introduction

Making research data available for reuse is an essential component for repeatable research [1]. Sharing data generated through publicly funded projects maximises return on investment and increases the likelihood of obtaining funding in future rounds [2]. Genomic data of human origin, when adequately shared, constitutes a direct measure of the current advancement in risk prediction, diagnosis, and treatment of genomic disorders [3]. Not only does human genome data have value to the individual, it is also of value to biological relatives of the individual, as well as to the wider research community, particularly when clinically actionable [4].

Sharing of human genomic data by researchers is governed by both legal and implicit obligations. Legal obligations include responsibilities and liabilities to protect the confidentiality and privacy of research participants, who, as data donors, intend and expect their data to be reused [5]. This is what is commonly referred to as the implicit ‘social contract’, which must be taken into account when developing the governance mechanisms around research participants. Although expectations from the social contract are implemented differently depending on local jurisdictions, for a concerned human genomics data generator (e.g., a researcher), it is important to be aware of local and international governmental regulations in place affecting the individual’s genetic data. In the United Kingdom, for example, there is currently a moratorium on the use of an individual’s genetic data for life insurance purposes [6]. In the United States, the Genetic Information Nondiscrimination Act (GINA) prevents employers from requesting, requiring, or purchasing the genetic information of their employees. GINA also prohibits health insurers acquiring genetic information for underwriting purposes and prior to enrolment [7]. In the European Union, there is no legislation specific to genetic information, although genetic data pertaining to health is considered ‘sensitive data’, and discrimination based on genetic features is prohibited [8]. This disparity among jurisdictions influences governance models for data providers who wish to maximise their data sharing in a FAIR (findable, accessible, interoperable, reusable) manner.

Sharing of human genomic data with external collaborators usually requires formal agreements and compliance with institutional review board (IRB) rules. An IRB may request the establishment of a data access committee (DAC) to regulate access to the data and define acceptable (re)use conditions. For researchers funded by international public funding bodies (e.g., National Institutes of Health [NIH] [9], Cancer Research UK [9,10], Wellcome Trust [11], Medical Research Council [MRC] [12], and others), it is common for investigators to share human genomic data broadly for secondary research purposes, in all cases, consistent with applicable laws, regulations, and policies.

Human genomic datasets are often accompanied by clinical phenotypes and other sensitive metadata, including pictures, medical history, sex, age, etc., building a picture of the patient to facilitate diagnostics and therapies. Despite a single genetic mutation datum itself not being a threat to the individual’s privacy, if whole genome data for an individual is made publicly available, removing direct identifiers (name, date of birth, etc.) may not be enough to conceal the identity of the individual. According to Homer et al. [13], it is straightforward to assess the probability that a person or relative participated in a study, especially if phenotype and clinical metadata are also available. However, the risk of re-identification may be mitigated. For example, Genomics England (GeL) [14] provides protected access (allowing authorised data users to access the de-identified data within the system) and enables export of only completely anonymised results. The consent framework implemented by GeL is thus in place for data to be accessible only to authorised users. Such solutions may work for specific research scenarios, yet commonly, researchers may require complete access to the research data.

Even when privacy risks are managed, an underlying problem may still remain: the systemic lack of standardised protocols for secure interoperability of genomic data globally. It is in particular this problem, combined with the lack of interoperable protocols and an increasing awareness of the need for human genomic data sharing, that led to the establishment of the Global Alliance for Genomics and Health (GA4GH) [15]. FAIR principles have been embraced by GA4GH and the community in general, providing a framework for data-sharing infrastructures [16]. FAIR principles are ideally suited to data repositories developing specialised strategies to facilitate the sharing of clinical data [17]. Examples of such data repositories include the European Genome-phenome Archive (EGA) [18] and the database of Genotypes and Phenotypes (dbGaP) [19]. Both store patient data of genetic and phenotypic origin, for which the patient has consented to reutilisation, approved for predetermined research uses via controlled data access. Specialised data journals such as *Scientific Data* [20], *GigaScience* [18], and *Human Genome Variation* [19] may also enforce best practices for publishing data whilst providing an incentive for researchers to share their data via a data paper.

Here, we propose five tips for providers of human genomic data wishing to use FAIR principles as a context of reference. We acknowledge that the act of sharing is a two-way process: the data producer may delegate the provision of the data to a trusted repository (data provider), where a data consumer finds and accesses the shared data. Our focus on human genomic data sharing from the data provider’s perspective originates as a consequence of developing Repositive [21], a global catalogue of human genomic data and metadata from data archives and repositories. Our mission and ongoing work to collate and connect the global landscape of data sources and datasets for genomics through an intuitive platform like Repositive has given us insight into common practices, enabling us to contribute to the discussion on how to maximise the FAIRness of shared human genomic datasets.

This is a *PLOS Computational Biology* Education paper.

### Tip 1: Establish a FAIR-aware patient consent framework

Consent frameworks dictate the extent to which human genomic data can be accessed and reused. Ensuring appropriate consent to collect genotype, phenotype, and any other type of human data is achieved will usually be the responsibility of the principal investigator (PI) overseeing the study. Data archives and repositories will be required to check that the consent forms of deposited datasets specify the goals of the immediate project. It is essential to explicitly describe in clear terms if the data is intended to be shared beyond the current scope of the project (i.e., general research use). If wider data sharing is intended, the consent form should set out potential risks and benefits to participants, as well as any data anonymisation procedures to be undertaken. Consent frameworks require special considerations from the data producer’s point of view, given their extreme variability. To allow standardisation of consent frameworks, GA4GH has developed consent codes that facilitate the integration of distinct consent types across different legal systems [22].

The level of anonymisation that will be applied to the data should be clearly explained in consent forms, since different levels are possible. Participant consent requirements should be considered prior to data collection, alongside approval from an IRB. Different research questions may necessitate variable degrees of identity exposure by study participants. For example, the Personal Genomes Project (PGP) provides complete access to study participants’ identities and phenotypic traits [23] under a Creative Commons Zero (CC0) license waiver [24]. This radically open consent framework is, however, a highly unusual one for clinical genomic data. NIH-funded studies require third-party researchers to submit a Data Access Request describing how they intend to use the data. A Data Use Certification Agreement is then produced, which must adhere to the NIH Genomic Data Sharing Policy’s ethical principles governing data access and privacy safeguards [25]. In the UK, GeL consent forms are classified according to whether patients are affected with cancer or rare diseases, with the consent framework allowing access to summary statistics in a controlled environment to authorised users [26].

Patient data sharing consent frameworks vary country to country, funder to funder, and study to study. We thus suggest that, for interoperability purposes, data sharing consent frameworks adopt existing standards for digital consent formats and include, at a minimum:

1. Goals of the current research project and why data generation/sharing is being carried out.
2. Potential risks to the individual participant from the (ab)use of the data.
3. Confirmation that these issues have been discussed in person, with the individual and/or guardian involved in signing the form.
4. Contract of data access for the current research project and the extent to which the data custodian commits to make the data findable, accessible, interoperable, and reusable for future research projects.

Some consent forms (e.g., PGP or Genomes Unzipped [27]) may make the patient/donor’s identity known. Others require the identity of research participants anonymised. The Database of Chromosomal Imbalance and Phenotype in Humans using Ensembl Resources (DECIPHER) database [28], a provider of anonymised human copy number variation (CNV) data and phenotypes (not datasets), offers a consent framework compliant with European Union guidelines for clinical sharing [29], allowing anonymous sharing of genomic and phenotypic data of patients. At all events, it is always advised that ethical and genetic counselling experts are consulted when choosing the appropriate consent form.

### Tip 2: Define FAIR data types specifying their intended uses and limitations

Providing sufficient information about the type of data being shared is fundamental for maximising data reuse. Deciding on their shared data and metadata descriptions also affects how datasets are found, accessed, and interoperated. Data consumers need to be able to find in the metadata what format the data is in and what size the files are as well as its provenance. It is also important to clearly define the technologies the data originated from, the experimental conditions, and any limitations as to how the data can be reused to ensure compliance with participants’ original consent forms. Phenotype or clinical history data is also essential for generating research outcomes. It may include controlled vocabularies or extensive free text. A number of controlled vocabularies such as the Human Phenotype Ontology [30] facilitate phenotypic annotation but offer no guarantee of having all needed fine-grained detail. Clinical history and further vital measurements may also vary according to study, instrument, or clinical need. Hence, extreme care must be ensured in establishing the procedure with which the data itself is to be transferred, e.g., ‘pretty good privacy’ encryption [31] or Aspera [32]. The choice for data transfer will be greatly influenced by the characteristics of the dataset, the consent framework, the amount of data to be accessed, and the repository where the data is stored.

For human genome–based data, it is important to make a distinction between raw and processed data types. Raw sequencing data must be processed before it can be interpreted. The processing of raw data is usually dependent on the software and parameters chosen to create interpretable data (e.g., variation calls). The choices of both software and parameters for processing raw data (and its intermediary files) are deeply influenced by the research questions being tested. Being able to capture the processing methodology in the metadata descriptions may be crucial for some experiments. Sometimes, however, researchers may choose not to redo the processing steps and simply reuse the interpretable data, but this might not be out of choice: the size of raw reads from whole human genome experiments can be prohibitively voluminous, depending on the coverage of the run. Therefore, the size of both the raw and processed data files to be shared will impact on the ease with which they can be reused. For example, variant call format (VCF) processed files [33] are much ‘lighter’ in storage footprint than binary alignment/map format (BAM) files [34] (at the cost of losing some information), so it may be better to provide Fastq files as raw data files from which BAM and VCF files can be derived.

### Tip 3: Maximise machine-readable data and metadata findability and interoperability

Maximising the likelihood for data to be found is a vital component of the data sharing process. For this, capturing of health/clinical data with complete, coherent, and standard descriptions is critical. The richness, granularity, and compliance to standards with which metadata descriptors are captured are determinant in influencing the user’s ability to reuse and draw any value from the dataset. A good template that incorporates specific pathophenotypic descriptions and patient annotations such as health constants, smoking status, clinical history, etc., is the PGP-Harvard data collection [23] and its accompanying raw and processed human genome data. This ideal level of data and metadata capture may not be attained by the sometimes constrained experimental conditions, data access options, or consent frameworks. The investigation, study, and assay (ISA) modelling tool may help guide experimental metadata collection [35] using the BioSharing (now FAIRsharing) catalogue of known standards [36]. The use of the minimum information about a microarray experiment (MIAME) standard for microarray data [37], for example, increases the discoverability of microarray data in MIAME-compliant repositories such as National Center for Biotechnology Information’s (NCBI) Gene Expression Omnibus (GEO) [38] and European Bioinformatics Institute’s (EBI) ArrayExpress [39].

Despite rich, standard metadata capture increasing the interoperability of datasets in any given repository, there is the additional problem of an increasing number of data repositories existing around the world. Thus, a number of metadata catalogues have been created to increase the discoverability of datasets. These catalogues include Repositive [40], DataMed [41], and OmicsDI [42], all with different aims and scope. Such meta-indexing solutions make it even more critical to standardise metadata descriptions.

Apart from (a) the great hurdle of scattered data sources, (b) different regulatory frameworks, and (c) heterogeneity of data types, there is the problem of how to incentivise scientists to contribute to the best possible standard of metadata annotation. Arguably, producers/generators of data are disincentivised to share as much data as possible in a standard, coherent, and complete manner, as the perceived risks are high and their rewards may be few. Making the data shareable means that the producers of data can be scrutinised by the community. Allowing the data to be discoverable, accessible, and reusable may also increase the risk that others will reap the reward of the effort of making the data shareable. To counteract these disincentives, data producers may allow early access to their data if they retain the privilege of publishing it first. Data papers may also increase the findability of the dataset, since the article will be indexed in bibliographic databases such as PubMed, where more discoverability of the dataset can be attained. Publishing discoverable, citable data increases the amount of citations for scientific output, resulting in greater incentive for FAIR data publishing as a measure of acknowledgment for work and scientific merit [43].

It is worthwhile to plan for ways in which the data itself can be made discoverable, as well as considering both the human and machine accessibility of the dataset. For example, it was recently shown that human gene symbols were converted to dates in the supplementary data files of some published papers [37], which meant these gene symbols were not machine readable. This is an issue particularly when data is not part of the manuscript review process in a publishing context, i.e., data validation checks are not in place (as opposed to automated/manual checks in databases). Simple strategies can be used to avoid these errors, such as including data units in tables and keeping data types consistent across columns or rows to avoid mixing of strings with numbers. It is best to avoid the use of acronyms where possible and to make sure they are defined if their use is unavoidable.

It is essential to ensure human genomic data is shared in a citable way. Data citations’ growing importance as a way to incentivise FAIR data sharing is being attested by the way in which researchers can gain recognition for making data available as well as providing provenance for it. A FAIR-aware data repository will enable data to be cited by providing a persistent and unique identifier for each data archive so that large-scale data interoperability is attained [44]. The main NCBI and EBI databases use accession identifiers, and other repositories may use DataCite’s Digital Object Identifiers (DOIs). Both accession IDs and DOIs can be cited in scholarly works, as in the guidance *Scientific Data* provides to its authors on how to cite data [45]. Similarly, scholarly works associated with a dataset should be referenced in the uniquely identified data record in the data archive: e.g., PubMed IDs might be added to the sequencing studies using either the interactive or programmatic route [46].

### Tip 4: Choose the most findable and accessible genomic data repository

The dataset type may impact on the type of repository that can be used to share the data. For example, the generalist repository figshare offers single file uploads of 5 terabytes per file but does not support controlled access to sensitive data. Using specialist data repositories for human genomic data may help ensure that this data is archived and preserved in a data type–specific way. For example, array-based human data would usually be submitted to repositories such as GEO [38] or ArrayExpress [46], while raw sequence data should usually go to repositories such as the Sequence Read Archive (SRA) [40] or the European Nucleotide Archive (ENA) [41]. Both SRA and ENA also store aligned data and data analysis (e.g., genome assemblies, taxonomic and gene class, etc.). For clinical genomic dataset deposition, the European Genome/phenome Archive (EGA) and the NCBI equivalent database of Genotypes and Phenotypes (dbGaP) are well-recognised controlled-access data archives. Both resources allow submission of sequencing, array-based data, and phenotypes as well. Care should be taken to archive controlled-access data in repositories that have workflows in place to ensure data access is only given to those requestors that fulfil the relevant consent requirements. Several funders have published lists of recommended repositories for specific types of research output (e.g., Wellcome Trust [47] and NIH [48]).

### Tip 5: Set FAIR data access governance

Data access governance is in great measure influenced by the consent framework (see Tip 1), the applicable jurisdiction, and the experimental design. The implementation of the data access policy will also be influenced by the technical strategy set in place. We expect that both technical and ethical/legal implementations will continue to evolve as types of human genomic data and their characteristics continue to change. Thus, a flexible approach is much needed. We turn again to GA4GH as a good guide for researchers and clinicians in choosing the right policy and technical implementation. We currently envisage a spectrum of access [49]. At one extreme, we have the open access approach with complete disclosure of the individual’s identity as exemplified by the PGP. At the other extreme, we have DAC-regulated access, in which access to data is subject to a contract between the user and the DAC, to be reviewed by the DAC and granted only if approved. In the case of dbGaP [50], the contract is signed with the US government, while for EGA, the contract is signed with the Wellcome Trust Sanger Institute. Both EGA and dbGaP act as the conduits that allow contract exchange implementation via their respective platforms. The access to the data is ultimately granted by the DAC. Access standards are not coordinated between international institutions, thus creating a huge overhead burden when data consumers require access from different studies across disparate data sources. There is at least (through GA4GH) an effort to facilitate the mutual recognition of independent separate DACs to save having to apply separately to multiple DACs when needing to access data from separate studies or independent sources.

The benefit of having regulated access to data via DACs is evident. With a DAC, every access request is evaluated against the consent given by patients and individual data donors, and the access to this data is provided only to intended recipients. The flip side of this is that undergoing the whole application process from dataset identification to data access can take time. Intermediary implementations of regulated access have been developed independently to avoid wasted time and effort applying to a DAC, reducing the likelihood of requesting access to the wrong type of dataset. GA4GH, for example, has developed the Beacon project, which allows standard programmatic querying of distributed sources for presence or absence of genetic features, given a dataset. The dbGaP Data Browser [51] has minimised the process of viewing general research use (GRU) data (currently 13% of all dbGaP subjects) to take less than 2 weeks. The dgGaP Data Browser reduces the number of unnecessary data downloads, allowing researchers to assess patient data before downloading it while decreasing chances for this data to be abused. Gaining download access to dbGaP data, however, still requires the submission of a dbGaP Project Request approval for each dataset [51].

Establishing the governance of access to shared datasets requires the awareness that once the data is acquired by the user, there is no easy way to track the usage of data by the data receiver. In any case, the DAC can always be utilised as the point of contact should there be need for consent frameworks to be modified given the evolving nature of research questions. Similarly, Data Access Agreements specify the custodianship of the data as well as the exceptional requirements of reporting incidental findings or what to do when inadvertently identifying an individual.

## Conclusion

Multiple funders and experts in data curation agree that sharing of personal health-related data must be planned from the start of the research project in order for it to be FAIR. Whenever it is possible to anonymise research data, this is the advised procedure for data producers to follow before data is shared. For data that has not been consented for open access, additional governance procedures for data access need to be established. For a compelling overview of all aspects to do with human genome data sharing, we direct our readers to [52].

This work contextualises current best practices for data providers assuming the role of dissemination agents for data producers. We specify that, in every sharing transaction of human genomic data, both a data consumer and a data provider are involved in establishing a secure data exchange. We embrace the FAIR data sharing principles described by Wilkinson et al. [16] and apply them to our particular ‘data provider’ context, which we have worked on as part of our wider efforts to catalogue the human genomic data landscape via the Repositive platform [21]. As precision medicine starts to impact patient lives, it is expected that sharing of datasets containing potentially sensitive information will become more widespread. Hence, having a set of guiding tips that help keep patient genomic data reusable whilst complying with consent frameworks is crucial if we are to leverage the power of FAIR principles to realise the promise of better diagnostics and more personalised therapies.
